# Metabolic and biochemical profile in women with polycystic ovarian syndrome attending tertiary care centre of central NEPAL

**DOI:** 10.1186/s12905-023-02379-z

**Published:** 2023-04-28

**Authors:** Isha Rajbanshi, Vijay Kumar Sharma, Eans Tara Tuladhar, Aseem Bhattarai, Mithileshwar Raut, Raju Kumar Dubey, Poonam Koirala, Apeksha Niraula

**Affiliations:** 1 Department of Clinical Biochemistry, Institute of Medicine, Tribhuvan University Teaching Hospital, Maharajgunj, Nepal; 2 Department of Obstetrics and Gynecology, Institute of Medicine, Tribhuvan University Teaching Hospital, Maharajgunj, Nepal

**Keywords:** Polycystic ovary syndrome/metabolism, Polycystic ovary syndrome*/diagnosis, Thyroid stimulating hormone, Body mass index, Dyslipidemia

## Abstract

**Background:**

Polycystic Ovarian Syndrome (PCOS) is a common endocrinopathy in women of reproductive age group and is highly associated with an increased risk of diabetes, hypertension, cardiovascular disease, and hyper estrogen-related malignancies in women with PCOS. This study was intended to assess the metabolic and hormonal profile of the patients with polycystic ovarian syndrome attending a tertiary care hospital.

**Methodology:**

A descriptive cross-sectional study was conducted among 107 women diagnosed with polycystic ovarian syndrome from the Department of Clinical Biochemistry of Tribhuvan University and Teaching Hospital. Descriptive analysis was performed to determine the socio-demographic characteristics of the participants. Bivariate analysis was conducted to determine using a t-test for comparing means between two groups and ANOVA for comparing the hormonal and metabolic parameters.

**Results:**

The mean age of the participants was 27 ± 4 years. This study showed that blood pressure was significantly higher in overweight and obese women (p = 0.001). The obese group had significantly higher serum TSH than the normal group (10.04 vs. 2.73, p = 0.001). Abnormal glucose and hyperinsulinemia were present in 4% of the patients, while 40% had Vitamin D deficiency. Hypothyroidism (TSH ≥ 4.5 mIU/ml) was found in 11% of the PCOS participants with a mean value of 6.65 ± 21.17 mIU/ml. Hyperprolactinemia ≥ 26.8 ng/ml was depicted in 21% of the study population with a mean value of 37.25 ± 21.86 ng/ml.

**Conclusion:**

Our study demonstrated that PCOS is most commonly prevalent in young women of the reproductive age group which can lead to reproductive, metabolic, and oncological complications in the long term. LH/ FSH ratio was found to be significantly deranged indicating that PCOS should be diagnosed and treated early in the adolescent age group.

## Introduction

The most common endocrinopathy in women of reproductive age is a polycystic ovarian syndrome (PCOS), with a prevalence of up to 7 to 10% [1]. It is a heterogeneous endocrine disorder characterized by polycystic ovaries, hyperandrogenism, and irregular menstrual cycles. Diagnosis of PCOS is made if two of the three criteria of androgen excess, ovulatory dysfunction, or polycystic ovaries are met [2]. The development of PCOS is significantly influenced by a variety of genetic and environmental variables. Risk factors for the onset of PCOS include obesity, lack of physical exercise, family history, and diabetes mellitus [3, 4]. Women with PCOS have a higher risk of developing insulin resistance, hypertension, psychiatric disorders, lipid disorders, diabetes, cancer, and osteoporosis. With a prevalence of 0.6 to 3.4% in infertile couples, PCOS is increasingly shown to be a significant factor in female infertility. In recent years, with improving laboratory facilities, sonography, and routine laparoscopic evaluation of infertility, PCOS has shown a remarkable increase in incidence. Due to the increased risk of diabetes, hypertension, cardiovascular disease, and hyperestrogen-related malignancies in women with PCOS, it needs thorough evaluation and treatment [5]. This study was intended to assess the metabolic and hormonal profile of the patients with polycystic ovarian syndrome attending a tertiary care hospital.

### Methodology

This descriptive cross-sectional study was carried out in the Department of Clinical Biochemistry in collaboration with the Department of Obstetrics and Gynecology Out-patient Department (OPD) (Infertility clinic) of Tribhuvan University Teaching Hospital (TUTH), Maharajgunj, Kathmandu with the time frame of six months. Ethical clearance was taken from Institutional Review Committee on November 11, 2021, [Reference No. (177(6–11) E2)]. PCOS patients were selected based on Rotterdam’s (2003) criteria [2]. For diagnosis, two of the following three criteria were required: Oligo- or anovulation, hyperandrogenism, and polycystic ovaries.

The prevalence was taken from the study of Vaidya et al. [2] and the sample size was calculated using Cochran’s formula,

n= (Z^2^ x p x (1-p)/e^2^

= (1.962 x 0.07 x 0.93)/0.052 = 100

Where Z = 1.96 (At 95% confidence interval) p = prevalence 7%.

Convenient sampling was used to enroll the patients diagnosed with PCOS visiting the infertility clinic of TUTH. Female patients aged 18–36 years diagnosed with PCOS who visited the Gynecology OPD of TUTH were included. Patients who were under medications like oral contraceptive pills, anti-uricaemic drugs, and anti-thyroid drugs and those who didn’t provide consent were excluded from the study. Written informed consent was taken from every study participant. A pre-structured proforma was used to obtain the clinical history and other patient-related details. Relevant clinical details of each participant were noted. Height and weight were measured and body mass index (BMI) was calculated as kg/m^2^. Weight gain and obesity contribute to the development of PCOS. World Health Organization (WHO) and National Institute of Health (NIH) criteria were used to classify BMI < 18.5 as underweight, BMI between 18.5 and 24.9 as normal weight, BMI between 25 and 29.9 as overweight and obesity with BMI > 30. Blood pressure measurement was done following the standard protocol using a mercury sphygmomanometer (Heine Optotechnik GMBH and Co. Kg, Germany) and a standard stethoscope. The blood sample was collected following standard phlebotomy protocol and analysis was done in the fully automated Enhanced Chemiluminescence Immunoanalyzer E-CLIA, Ortho Diagnostics (Johnson and Johnson). All experiments were performed in accordance with relevant guidelines and regulations.

Statistical analysis was done by Statistical Package for Social Sciences (SPSS 22 version). Descriptive statistics like Mean, Standard Deviation, and Median were used to represent the socio-demographic data. Mean comparisons were done by independent t-test. Group association was determined using the Chi-square test. The correlation was determined by Spearman’s correlation. ANOVA was used for the comparison of hormonal parameters in 3 groups: normal, overweight, and obese patients with PCOS. Multiple linear regression was used to find out the association of metabolic and hormonal parameters in obese PCOS patients. The level of significance was set at a p-value < 0.05.

## Results

Our study enrolled 107 women diagnosed with polycystic ovarian syndrome. The mean age of the patients was 27 ± 4.09 years. The majority of the patients (56.07%) were between 25 and 30 years (Table 1¸2). The mean systolic blood pressure was 114.95 ± 12.08 mmHg and diastolic blood pressure was 67.11 ± 6.91 mmHg.

**Table 1:** Sociodemographic and clinical characteristics of the participants

Variables	Value (Mean ± S.D.)	Frequency (n = 107)
Age (years)	27.10 ± 4.09	
18–24 years		19 (18%)
25–30 years		60 (56%)
31–36 years		28 (26%)
**BMI** (kg/m^2^)	24.81 ± 3.71	
Normal		57 (53%)
Overweight		40 (37%)
Obese		10 (9%)
SBP (mm Hg)	114.95 ± 12.08	
DBP (mm Hg)	67.11 ± 6.91	

65% of the participants were from the hilly region, 29% were from the Terai and 6% were from the mountain region. The mean Body Mass Index (BMI) was (24.4 ± 3.55 kg/m^2^). Among them, 37% were overweight and 9% were obese as depicted in Table 1.

**Fig. 1:**
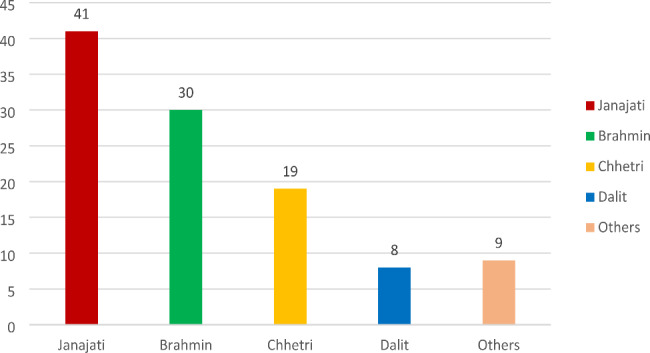
Distribution of PCOS cases based on ethnicity

The distribution of the patients as per ethnicity depicted that the majority of the PCOS patients were Janajatis (38%) followed by Brahmins (28%), Chettris (18%), Dalits (7.5%), and others (8.5%) as shown in Fig. 1.

Assessment of hormonal parameters showed that (19%) of the PCOS patients had LH/FSH ratio ≥ 2. The mean hormonal value of prolactin was 17.80 ± 15.89 ng/ml. The mean TSH value was 2.50 ± 7.70 mIU/ml. The testosterone level of PCOS participants was 35.8 ± 20.15 ng/ml whereas the progesterone level was 1.73 ± 21.73 ng/ml. The mean estrogen level was 31.35 ± 19.78 pg/ml. The mean value of glucose was 5.0 ± 0.86 mmol/l and vitamin D was 22.6 ± 7.92 ng/ml. Mean HDL level was 0.95 ± 0.13 mg/dl, LDL level was 4.0 ± 0.86 mg/dl, TC 5.25 ± 0.83 mg/dl and TG was 1.1 ± 0.35 mg/dl as shown in Table 2.

**Table 2:** Biochemical parameters in the study population

Variables	Values
fT3	5.3 ± 1.65
fT4	13.4 ± 5.5
TSH	2.5 ± 7.7
FSH	6.20 ± 2.19
LH	7.09 ± 5.5
Prolactin	17.8 ± 15.89
Estrogen	31.35 ± 19.78
Progesterone	1.73 ± 21.73
Testosterone	35.8 ± 20.15
Vitamin D	22.2 ± 7.63
Glucose	5.0 ± 0.766
HDL	0.95 ± 0.12
LDL	4.0 ± 0.86
TC	5.25 ± 0.82
TG	1.1 ± 0.34

Comparison of the hormonal parameters on the basis of BMI demonstrated that age, vitamin D, fT3, FSH, and LH/FSH ratios were comparable in all the 3- normal weight, overweight, and obese groups. However, our study depicted blood pressure to be significantly higher in the overweight and obese group compared to the normal (p = 0.001). The obese group had significantly higher serum TSH than the normal group (10.04 vs. 2.73, p = 0.001) as depicted in Table 3.

**Table 3:** Comparison of hormonal parameters in normal, overweight and obese patients with PCOS

Variables	Normal (< 25 kg/m^2^)	Overweight (25–30 kg/m^2^)	Obese (> 30 kg/m^2^)	p value
Age	27.44 ± 3.8	25.68 ± 3.77	28.38 ± 5.02	0.039*
SBP	109.81 ± 10.54	121.63 ± 9.76	120.77 ± 14.97	0.001*
DBP	64.65 ± 7.27	71.50 ± 7.86	71.15 ± 9.16	0.001*
Glucose	5.0 ± 0.69	5.01 ± 0.95	5.39 ± 1.16	0.32 ^a^
Vitamin D	23.10 ± 8.19	22.61 ± 8.10	23.03 ± 6.72	0.96^a^
fT3	5.35 ± 1.67	5.16 ± 1.45	5.49 ± 2.15	0.78 ^a^
fT4	13.18 ± 5.35	11.64 ± 5.29	9.97 ± 6.28	0.11 ^a^
TSH	2.73 ± 1.53	2.71 ± 1.37	10.04 ± 21.39	**0.005** ^**a**^ *
FSH	6.77 ± 2.16	6.33 ± 2.06	6.63 ± 2.16	0.60 ^a^
LH	8.4 ± 5.74	8.29 ± 5.15	8.06 ± 6.14	0.98 ^a^
LH/FSH	1.38 ± 1.17	1.26 ± 0.63	1.03 ± 0.28	0.68 ^a^
Prolactin	22.14 ± 18.95	22.94 ± 12.56	15.51 ± 9.15	0.33 ^a^
Oestrogen	31.60 ± 16.83	38.69 ± 23.82	33.15 ± 16.19	0.22 ^a^
Progesterone	6.94 ± 8.48	5.43 ± 7.43	3.01 ± 5.64	0.24 ^a^
Testosterone	39.49 ± 20.29	42.18 ± 21.26	33.59 ± 15.40	0.41 ^a^

a = ANOVA; *p value < 0.05 is considered to be statistically significant

Metabolic parameters of the study population demonstrated that abnormal glucose ≥ 7.8 mmol/l was seen in 4% of the patients and vitamin D deficiency was depicted in 40% of the participants as described in Table 4.

**Table 4:** Metabolic parameters in PCOS patients

Variables		Values	Mean ± S.D.
BMI	< 25 kg/m^2^	59 (55%)	23.07 ± 2.01
	> 25 kg/m^2^	48 (45%)	27.0 ± 2.79
Glucose	< 7.8 mmol/l	103 (96%)	5.0 ± 0.51
	≥ 7.8 mmol/l	4 (4%)	8.3 ± 0.44
Vitamin D	≤ 20 ng/ml	43 (40%)	16.7 ± 4.25
	20–30 ng/ml	50 (47%)	24.95 ± 2.74
	≥ 30 ng/ml	14 (13%)	35.95 ± 4.54

Hypothyroidism (TSH ≥ 4.5 mIU/ml) was found in 11% of the PCOS participants with a mean value of 6.65 ± 21.17 mIU/ml. Hyperprolactinemia ≥ 26.8 ng/ml was depicted in 21% of the study population with a mean value of 37.25 ± 21.86 ng/ml. 3% of participants were found to have increased estrogen > 75 pg/ml with a mean value of 114.79 ± 21.94 pg/ml. 75% of the participants had low progesterone levels ≤ 10.8 ng/ml with a mean value of 1.68 ± 1.18 ng/ml. High testosterone > 70 ng/ml was depicted in 7% of the PCOS women as shown in Table 5.

**Table 5:** Hormonal parameters in PCOS patients

Variables		Values	Mean ± S.D.
TSH	≤ 4.5 mIU/ml	95 (89%)	2.2 ± 0.97
	≥ 4.5 mIU/ml	12 (11%)	6.65 ± 21.17
Prolactin	≤ 26.8 ng/ml	84 (79%)	15.55 ± 6.01
	≥ 26.8 ng/ml	23 (21%)	37.25 ± 21.86
Estrogen	< 25 pg/ml	32 (30%)	16.09 ± 6.71
	25–75 pg/ml	72 (67%)	38.25 ± 9.95
	> 75 pg/ml	3 (3%)	114.79 ± 21.94
Progesterone	≤ 10.8 ng/ml	80 (75%)	1.68 ± 1.18
	≥ 10.8 ng/ml	27 (25%)	19.7 ± 5.44
Testosterone	≤ 70 ng/ml	100 (93%)	35.15 ± 15.89
	> 70 ng/ml	7 (7%)	82.9 ± 15.89

Spearman’s correlation for metabolic and hormonal parameters in the study population showed that LH was negatively correlated with age, LH/FSH ratio, and estrogen with the correlation being statistically significant. Serum glucose was negatively correlated with vitamin D levels. Similarly, there was a significant negative correlation between TSH and LH, LH/FSH ratios respectively. Also, LH/FSH ratio was significantly correlated with E2 levels in PCOS patients. Spearman’s correlation for metabolic and hormonal parameters in obese PCOS patients revealed that vitamin D was negatively correlated with estrogen levels in obese PCOS patients. Similarly, testosterone was positively correlated with glucose levels, while there was a significant negative correlation between testosterone and LH/FSH ratio, and Prolactin respectively (p < 0.05) as shown in Table 6.

**Table 6:** Spearman’s correlation for metabolic and hormonal parameters in the study population

Variables	Age	BMI	Glucose	Vitamin D	fT3	fT4	TSH	FSH	LH	LH/FSH	PRL	E2	Progesterone	Testosterone
Age	-	r = 0.03 p = 0.73	r = 0.04 p = 0.64	r = 0.03 p = 0.75	r= -0.09 p = 0.35	r= -0.19* p = 0.05	r= -0.12 p = 0.23	r= -0.09 p = 0.93	r= -0.24* p = 0.01	r= -0.26* p = 0.01	r = 0.04 p = 0.69	r= -0.21* p = 0.03	r= -0.03 p = 0.77	r = 0.03 p = 0.74
BMI	r = 0.03 p = 0.73	-	r = 0.09 p = 0.36	r = 0.02 p = 0.80	r=-0.08 p = 0.42	r= -0.17 p = 0.09	r= -0.09 p = 0.35	r= -0.16 p = 0.11	r= -0.13 p = 0.18	r= -0.01 p = 0.87	r= -0.08 p = 0.42	r = 0.07 p = 0.49	r= -0.02 p = 0.82	r= -0.06 p = 0.52
Glucose	r = 0.04 p = 0.64	r = 0.09 p = 0.36	-	r= -0.22* p = 0.02	r = 0.01 p = 0.99	r= -0.01 p = 0.86	r = 0.10 p = 0.28	r= -0.10 p = 0.32	r= -0.10 p = 0.31	r= -0.16 p = 0.10	r = − 0.04 p = 0.66	r= -0.06 p = 0.55	r = 0.14 p = 0.14	r = 0.03 p = 0.72
Vitamin D	r = 0.03 p = 0.75	r = 0.02 p = 0.80	r= -0.22* p = 0.02	-	r = 0.06 p = 0.95	r= -0.08 p = 0.43	r= -0.13 p = 0.18	r= -0.14 p = 0.16	r= -0.04 p = 0.97	r=-0.04 p = 0.66	r= -0.02 p = 0.77	r= -0.06 p = 0.53	r = 0.08 p = 0.41	r=-0.11 p = 0.26
fT3	r= -0.31* p = 0.03	r=-0.08 p = 0.42	r = 0.01 p = 0.99	r = 0.06 p = 0.95	-	r = 0.46* p = 0.001	r= -0.07 p = 0.46	r = 0.06 p = 0.50	r = 0.02 p = 0.84	r= -0.02 p = 0.84	r = 0.03 p = 0.70	r= -0.03 p = 0.72	r= -0.06 p = 0.53	r = 0.03 p = 0.73
fT4	r= -0.19* p = 0.05	r= -0.17 p = 0.09	r= -0.01 p = 0.86	r= -0.08 p = 0.43	r = 0.46* p = 0.001	-	r=-0.04 p = 0.69	r = 0.15 p = 0.12	r = 0.12 p = 0.23	r = 0.06 p = 0.52	r = 0.29** p = 0.004	r = 0.95 p = 0.35	r=-0.11 p = 0.25	r = 0.11 p = 0.27
TSH	r= -0.04 p = 0.79	r= -0.12 p = 0.23	r = 0.10 p = 0.28	r= -0.13 p = 0.18	r= -0.07 p = 0.46	r=-0.04 p = 0.69	-	r = 0.14 p = 0.15	r= -0.16 p = 0.10	r=-0.21* p = 0.03	r=-0.05 p = 0.58	r = 0.14 p = 0.15	r= -0.06 p = 0.54	r=-0.15 p = 0.14
FSH	r= -0.09 p = 0.93	r= -0.16 p = 0.11	r= -0.10 p = 0.32	r= -0.14 p = 0.16	r = 0.06 p = 0.50	r = 0.15 p = 0.12	r = 0.14 p = 0.15	-	r = 0.24* p = 0.01	r= -0.20* p = 0.04	r = 0.11 p = 0.25	r = 0.13 p = 0.20	r = 0.01 p = 0.89	r= -0.07 p = 0.46
LH	r= -0.24* p = 0.01	r= -0.13 p = 0.18	r= -0.10 p = 0.31	r= -0.04 p = 0.97	r = 0.02 p = 0.84	r = 0.12 p = 0.23	r= -0.16 p = 0.10	r = 0.24* p = 0.01	-	r = 0.86** p = 0.001	r = 0.17 p = 0.08	r = 0.30** p = 0.002	r= -0.14 p = 0.15	r = 0.13 p = 0.18
LH/FSH	r= -0.26* p = 0.01	r= -0.01 p = 0.87	r= -0.16 p = 0.10	r=-0.04 p = 0.66	r= -0.02 p = 0.84	r = 0.06 p = 0.52	r=-0.21* p = 0.03	r= -0.20* p = 0.04	r = 0.86** p = 0.001	-	r = 0.13 p = 0.18	r = 0.27** p = 0.006	r= -0.12 p = 0.21	r=-0.16 p = 0.09
Prolactin	r = 0.04 p = 0.69	r= -0.08 p = 0.42	r = − 0.04 p = 0.66	= -0.02 p = 0.77	r = 0.03 p = 0.70	r = 0.29** p = 0.004	r=-0.05 p = 0.58	r = 0.11 p = 0.25	r = 0.17 p = 0.08	r = 0.13 p = 0.18	-	r = 0.06 p = 0.53	r= -0.23* p = 0.02	r = 0.02 p = 0.83
Estrogen	r= -0.21* p = 0.03	r = 0.07 p = 0.49	r= -0.06 p = 0.55	r= -0.06 p = 0.53	r= -0.03 p = 0.72	r = 0.95 p = 0.35	r = 0.14 p = 0.15	r = 0.13 p = 0.20	r = 0.30** p = 0.002	r = 0.27** p = 0.006	r = 0.06 p = 0.53	-	r= -0.06 p = 0.53	r= -0.12 p = 0.22
Progesterone	r= -0.03 p = 0.77	r= -0.02 p = 0.82	r = 0.14 p = 0.14	r = 0.08 p = 0.41	r= -0.06 p = 0.53	r=-0.11 p = 0.25	r= -0.06 p = 0.54	r = 0.01 p = 0.89	r= -0.14 p = 0.15	r= -0.12 p = 0.21	r= -0.23* p = 0.02	r= -0.06 p = 0.53	-	r= -0.09 p = 0.36
Testosterone	r = 0.03 p = 0.74	r= -0.06 p = 0.52	r = 0.03 p = 0.72	r=-0.11 p = 0.26	r = 0.03 p = 0.73	r = 0.11 p = 0.27	r= -0.15 p = 0.14	r= -0.07 p = 0.46	r = 0.13 p = 0.18	r= -0.16 p = 0.09	r= -0.02 p = 0.83	r= -0.12 p = 0.22	r= -0.09 p = 0.36	-

a = Spearman’s correlation; *p-value < 0.05 is considered to be statistically significant

Multiple regression analysis showed that the estrogen level was significantly associated with abnormal BMI in PCOS patients as illustrated in Table 7 respectively.

**Table 7:** **Multiple linear regression of metabolic and hormonal parameters in obese (BMI ≥ 30 kg/m** ^**2**^ **) PCOS patient**

Variables	Coefficient	SE	t value	p value
Intercept	1.99	0.62	-	-
Age (years)	-0.07	0.01	-0.67	
Glucose	0.07	0.07	0.66	0.51
Vitamin D	0.04	0.007	0.37	0.71
fT3	-0.10	-0.04	-0.74	0.46
fT4	-0.13	-0.01	-0.93	0.35
TSH	0.14	0.007	1.30	0.19
FSH	-0.29	0.04	-1.68	0.09
LH	0.27	0.02	0.87	0.38
LH/FSH	-0.43	0.16	-1.37	0.17
Prolactin	0.04	0.003	0.38	0.70
Oestrogen	0.21	0.003	1.94	0.05*
Progesterone	0.02	0.002	0.26	0.79
Testosterone	0.03	0.003	0.33	0.74

Coefficient: regression coefficient; SE: standard error. *p value < 0.05 is considered to be statistically significant

## Discussion

PCOS is probably the most prevalent endocrinological disorder affecting females and is the most common cause of menstrual disturbance during reproductive age. It is characterized by the presence of polycystic ovaries on ultrasound and/or clinical and biochemical signs of hyperandrogenism and/or oli-anovulation [6].

The mean age of the patient in our study was 27.0 ± 4 years with the most common age group being 25–30 years (56.7%). Our findings were similar to the study reported in Nepal by Vaidya A et al [2]. PCOS is reported to be more prevalent in younger ages (< 35) than among older women, which can be attributed to a physiological decline of the follicular cohort leading to a normalized ovarian ultrasonographic appearance with advancing age [7].

High BMI is one of the strong risk factors for cardiovascular and other metabolic disturbances in PCOS. We found that 37% of our study participants were overweight, with BMI of 25–30 kg/m2. The mean BMI was 24.4 ± 3.55 kg/m^2^. 9% of the PCOS patients were obese with BMI > 30 kg/m^2^. Our finding is similar to the study done by Vaidya et al. [2] where 30% were overweight and 11% were obese. In the study by Sadia et al. in Saudi Arabia [8], the mean BMI for overweight was 21.5 ± 1.8 kg/m2 and the mean BMI for obese was 30.3 ± 4.4 kg/m^2^ [8].

In our study, the mean systolic blood pressure was 120 ± 12.25 mmHg and diastolic blood pressure was 70 ± 8.19 mmHg. The absolute level of SBP and DBP were not remarkably high. The number of hypertensive patients in our study was few. In the study done by Mellembakken et al., the mean systolic blood pressure was 118 ± 9.6 mmHg in women with PCOS compared to 110 ± 7.56 mmHg in controls and diastolic BP was 74 ± 7 mmHg vs. 70 ± 5.5 mmHg (p < 0.001). BP ≥ 140/90 mmHg was observed in 11.1% [9]. The findings were in accordance with that there is a close relationship between BMI and BP. The mechanism underlying the increased prevalence of hypertension in PCOS has been linked to several factors such as hyperinsulinemia, hyperandrogenemia, and obesity [10].

Abnormal glucose was demonstrated in 4% of the PCOS participants. Post-binding defect in signal transduction is responsible for insulin resistance [11].This defect results from the impaired activity of the kinase receptor. Dyslipidemia with high TG and low HDL concentrations is frequently noticed [11]. A study conducted by Chunla He et al. [12] depicted that lower vitamin D levels were significantly correlated with markers of hyperglycemia, supporting a significant difference in serum vitamin D levels between women with and without PCOS. Chunla He et al. [12] suggested that a low vitamin D level is related to elevated levels of total cholesterol, LDL, glucose, triglycerides, and decreased HDL in women affected by PCOS [12]. In agreement with these findings, our study reported an inverse correlation of serum vitamin D concentrations with glucose (p = 0.029). Hypovitaminosis D (Vitamin D ≤ 20 ng/ml) was observed in 40% of the participants.

The vitamin D receptor (VDR) can be found across several tissues within the female reproductive system. Calcitriol (1,25[OH]2 vitamin D) directly leads to the production of estrogen and progesterone, thus possibly potentiating granulosa cell luteinization and leading to an improved endometrial environment. Vitamin D suppresses parathyroid hormone secretion, adaptive immune response, and cell proliferation while promoting insulin secretion and innate immune response and stimulating cell differentiation. Vitamin D’s basic role in modulating different immunological properties, including lymphocyte activation and proliferation, differentiation of TH lymphocytes, production of specific antibodies, and regulation of the immune response. Vitamin D influences glucose homeostasis and insulin sensitivity [13].

The proportion of hypothyroidism in our study was 11% and the mean TSH level was 6.65 ± 21.17 mIU/L. A study by Fupare et al. [14] reported that hypothyroidism was present in 18% of the infertile women, which was significantly higher than in control groups [14]. Similarly, Kumkum et al. [15], reported that the incidence of hypothyroidism in hyperprolactinemic women was 25.50%. The present study depicts that there was no significant correlation between prolactin and TSH. The deficiency of thyroid hormones affects gonadal function and fertility, leading to delayed puberty onset and anovulatory cycles. Hypothyroidism in PCOS is associated with increased body weight, increased sex hormone-binding globulin (SHBG), androstenedione to testosterone conversion increase, and aromatization to estradiol [16].

A study by Davoudi et al. [17] manifested hyperprolactinemia in 37% of patients with a mean serum prolactin level of 48.42 ± 5.44 ng/ml [17]. In our study, hyperprolactinemia was observed in 21% of PCOS patients with a mean value of 37.25 ± 21.86 ng/ml which was lower than the study by Davoudi et al. [17]. It has been suggested the possibility of acceleration of GnRH pulsatility in PCOS women could be involved in the increase of LH and in the decrease of dopaminergic tone (which induces hyperprolactinemia) [18].

The normal gonadotrophin axis is disturbed in PCOS women. Therefore, LH levels increase, and FSH levels decrease, leading to a reversal of the LH/FSH ratio. 19% of the PCOS patients in our study had LH/FSH ratios ≥ 2. A higher incidence of increased LH/FSH ratio was observed in the study by Vaidya A et al., who depicted that 83% of the population with PCOS had a high LH/FSH ratio ≥ 2. A study by Alnakash et al. [19] depicted that there was no significant statistical correlation between BMI and LH/FSH ratio (r = 0.014, P > 0.05) [19].

Similarly, Saadia et al. reported no significant correlation between BMI and LH/FSH ratio which is concordant with our study [8]. Our study depicted a higher serum testosterone level > 70 ng/ml in 7% with a mean value of 82.9 ± 15.89 ng/ml. Similarly, the high testosterone level was depicted in the high BMI group compared with the normal BMI group (0.54 vs. 0.38 ng/ml, p = 0.044) in the study done by Saadia et al [8].

## Conclusion

The findings from our study depict that PCOS has varying metabolic effects like increased BMI, impaired glucose levels, low vitamin D, and hormonal alterations of fT3, fT4, TSH, FSH, LH, ER, progesterone, and testosterone. Our study demonstrated that PCOS is most commonly prevalent in young women of the reproductive age group and it has shown an association with long-term reproductive, metabolic, and oncologic complications. LH/ FSH ratio was found to be significantly deranged indicating that PCOS should be diagnosed and treated early in adolescence.

## Data Availability

The data analyzed in this study are available from the corresponding author on reasonable request.
